# Tactical Situations and Playing Styles as Key Performance Indicators in Soccer

**DOI:** 10.3390/jfmk9020088

**Published:** 2024-05-21

**Authors:** Spyridon Plakias, Themistoklis Tsatalas, Vasileios Armatas, Dimitris Tsaopoulos, Giannis Giakas

**Affiliations:** 1 Department of Physical Education and Sport Science, University of Thessaly, 42150 Trikala, Greece; spyros_plakias@yahoo.gr (S.P.); ttsatalas@uth.gr (T.T.); 2 School of Physical Education and Sport Science, National and Kapodistrian University of Athens, 10679 Athens, Greece; v-armatas@phed.uoa.gr; 3 Center for Research and Technology Hellas, 60361 Volos, Greece; dtsaop@gmail.com

**Keywords:** football, tactics, game style, performance analysis

## Abstract

The game of soccer is complex and unpredictable, demanding multifaceted strategies for success. Performance analysis has evolved, focusing on key performance indicators (KPIs) to determine the factors that most significantly influence a team’s success or failure during matches. Traditional performance analysis methods have emphasized quantifiable data like physical exertion and basic play events but often neglected the subtler tactical dimensions that could significantly impact game outcomes. This study aimed to fill the gap in the current literature by creating a comprehensive framework that incorporates tactical situations as KPIs. The objective was to examine whether specific playing styles adopted by teams in various tactical situations and phases of the game could predict the outcome of matches. A dataset comprising all First Division Championship matches from 11 different European countries for the 2021–2022 season was analyzed. Variables representing tactical situations were correlated with match outcomes using a Generalized Estimating Equation framework. The model was specified with a binomial distribution and a logit link function. Statistical significance was determined using Wald χ^2^ tests with a significance level set at *p* < 0.05. The study’s findings revealed that possession style, counterattacking during offensive transitions, and a balanced aggressive defensive strategy significantly increase a team’s chances of victory. It also showed that successful teams tend to focus on central attacks, minimize crossing, and execute strategic plays that lead to final attempts on goal with minimal ball possession. The above findings demonstrate that adopting certain tactical approaches significantly influences soccer match outcomes, highlighting the importance of considering tactical aspects as KPIs.

## 1. Introduction

Soccer is inherently a chaotic game, making success a multifactorial issue [1,2]. For this reason, performance analysis is a constantly evolving field [3], with performance analysts increasingly occupying positions within coaching staffs, and academic research in this area continuously expanding [4,5]. For performance analysts, the ability to accurately analyze and understand key performance indicators (KPIs) stands as a critical challenge. According to Butterworth et al. [6], who differentiated performance indicators (PIs) from KPIs, this challenge revolves around identifying the factors that most significantly influence a team’s success or failure during matches. Solving this problem is crucial as it directly impacts team strategy, training focus, and in-game decisions [7].

Previous efforts to address this challenge have primarily focused on event or tracking data to quantify physical exertions (e.g., total distance, distances covered in different intensity zones) and basic play events (e.g., ball possession time, shots on goal, accurate passes) [8,9,10], while others incorporate situational variables such as the venue or the strength of the opponent team [11,12]. In particular, most research has emphasized events data to determine the factors that distinguish winning teams [13,14,15,16,17,18]. Others have also used tracking data to examine physical parameters [19,20,21]. It is noteworthy that some of the above studies [16,19] have incorporated situational variables, as the impact of context on performance is significant [22]. Additionally, in recent years, artificial intelligence, thanks to the ability to use large volumes of data and a large number of variables, has offered new possibilities in exploring factors that can predict a victorious outcome [23,24,25].

However, these approaches often overlook the nuanced tactical dimensions that play a crucial role in the game’s outcome [26]. As highlighted in their review titled “Identifying Soccer Teams’ Styles of Play: A Scoping and Critical Review,” Plakias et al. [27] note that several authors have investigated the effectiveness of playing styles, but only one study [28] attempted to link playing styles with outcomes (win, lose, or draw). However, this study only explored four playing styles that resulted from the combination of two tactical situations (high or deep press and elaborate or direct attack). Additionally, from all the above research, very few used data from more than one competition. Specifically, Evangelos, Aristotelis, Ioannis, Stergios and Foteini [14] used a sample of 64 matches from four different European leagues in the 2013–2014 season; Zhou, Zhang, Lorenzo Calvo and Cui [19] used data from six seasons of the Chinese Super League; and Fernández-Cortés, Gómez-Ruano, Mancha-Triguero, Ibáñez and García-Rubio [13] employed a dataset from seven seasons of the Spanish LaLiga.

Therefore, the literature still lacks a comprehensive framework that integrates tactical aspects as KPIs. According to Sarmento et al. [29], tactics should be an essential element in performance analysis. If a more effective solution to pinpointing and leveraging tactical situations and playing styles as KPIs were found, teams could significantly improve their tactical planning and execution, leading to more strategic gameplay and increased chances of victory.

Based on the gap identified in the literature, we hypothesized that analyzing football performance through the lens of tactical situations and playing styles would provide more nuanced and actionable insights than traditional KPIs. Furthermore, we employed an extensive dataset of First Division Championship matches across 11 European countries. This represents one of the most comprehensive analyses of tactical KPIs in football to date. We assumed that this approach would reveal the strategic underpinnings of football matches, offering a rich perspective on performance analysis. Therefore, the purpose of our study was to investigate whether the adoption of specific playing styles by teams in various tactical situations and phases of the game can influence the outcome of a match.

## 2. Material and Methods

### 2.1. Sample

The sample included all First Division Championship matches from 11 different European countries in the 2021–2022 season. For each match, there were 2 observations (one for each team). Only the regular season matches were used (play-off and play-out matches were removed). A total of 174 teams participated in the 11 championships, each of which played 22 to 38 matches. Finally, 5992 valid observations were used in the analysis. The details for the sample are available in the original research by Plakias et al. [30].

### 2.2. Procedure

**Figure 1:**
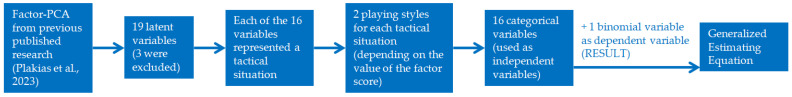
Flowchart illustrating the study procedure [30].

Of the 19 latent variables that emerged as factor scores in the original research by Plakias, Kokkotis, Moustakidis, Tsatalas, Papalexi, Kasioura, Giakas and Tsaopoulos [30], 16 were used as variables in the current study. Three of the nineteen were removed because they pertained to the game as a whole and not to one team specifically. Each of the 16 variables represented a tactical situation in which the team adopted a certain style of play. For each tactical situation, depending on the value of the factor score, the team preferred one of the two corresponding styles in each match. The 16 tactical situations along with the corresponding styles (depending on the factor score value) are shown in Table 1. Figure 1 illustrates the step-by-step process of our methodology.

### 2.3. Statistical Analysis

Data were analyzed using a Generalized Estimating Equation (GEE) framework to account for the correlated nature of the data with repeated measures (each team played 22–38 matches) [31,32]. The dependent variable was RESULT, coded as 1 (WIN) for the cases when the team won and 2 (NO WIN) for the cases when the team lost or drew. The independent variables included in the model are shown in Table 1. Due to the nature of the dependent variable, the model was specified with a binomial distribution and a logit link function. The working correlation structure was set as independent, because this gave the lowest values in the Quasi Likelihood under Independence Model Criterion (QIC) and Corrected Quasi Likelihood under Independence Model Criterion (QICC) when compared to the corresponding values obtained using the remaining working correlation structures [33]. Wald χ^2^ tests were used to assess the significance of the predictors, and results are presented with 95% confidence intervals. All analyses and graphical representations were performed using SPSS (version 25.00) software and the significance level was set at *p* < 0.05.

## 3. Results

**Figure 2:**
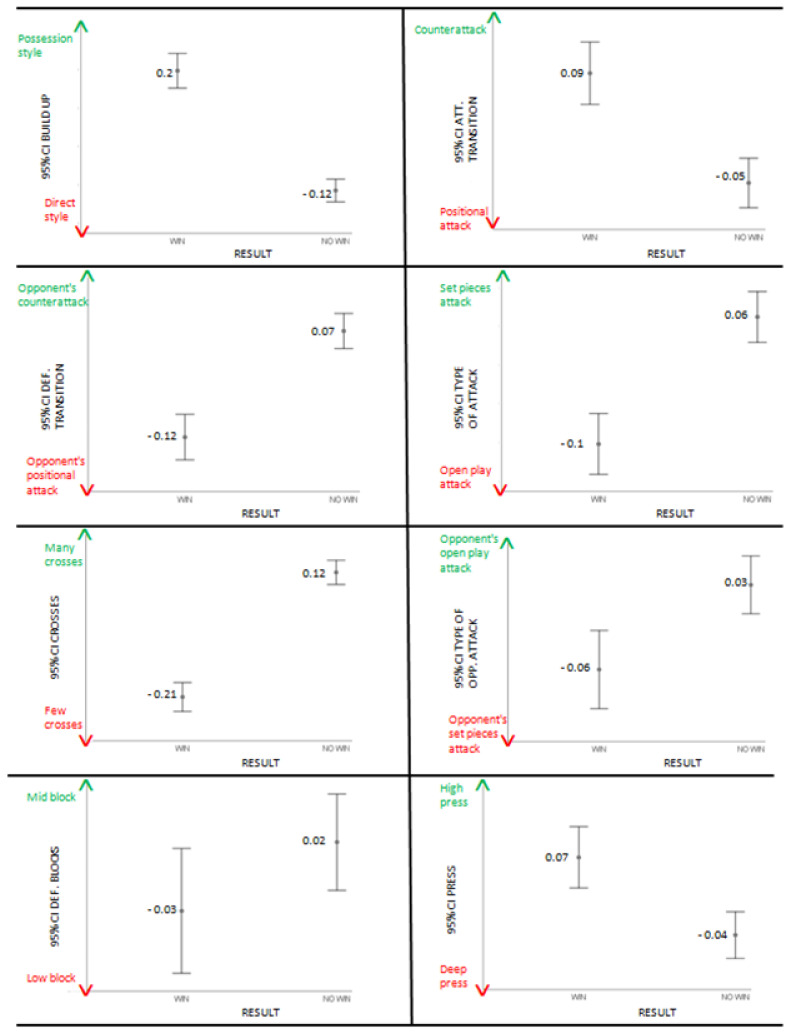
Error bars for build up, attacking transition, defensive transition, type of attack, crosses, type of opponent attack, defensive blocks, and press.

**Figure 3:**
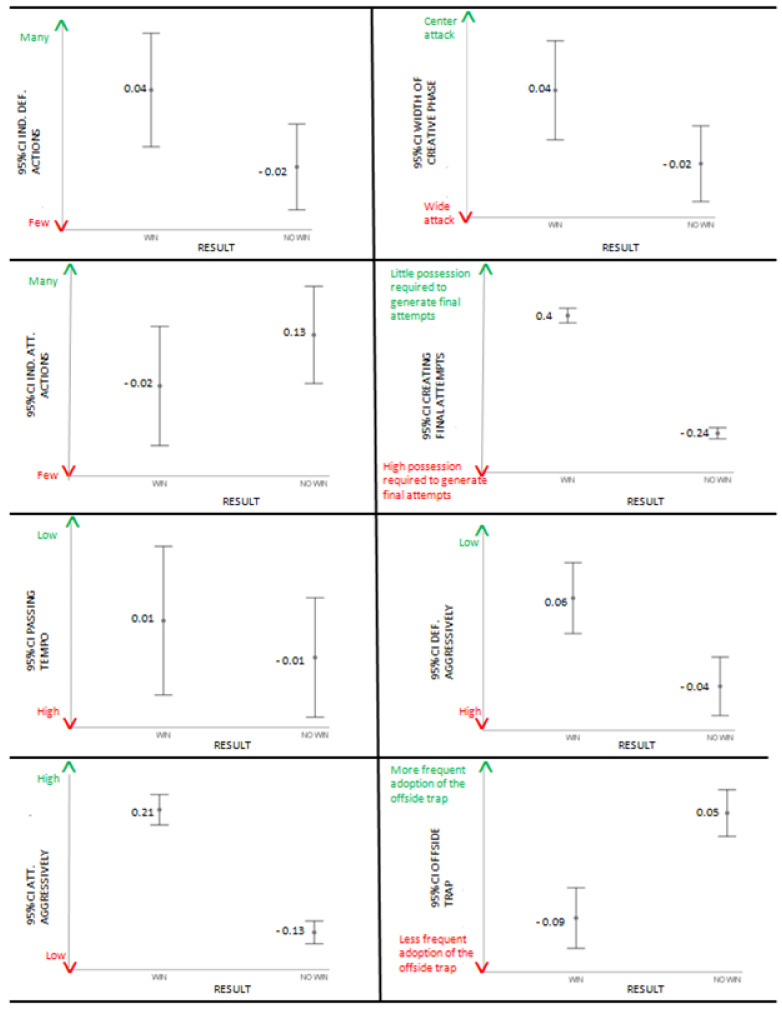
Error bars for individual defending actions, width of creative phase, individual attacking actions, creating final attempts, passing tempo, defending aggressively, attacking aggressively, and offside trap.

Table 2 shows that all variables significantly explain the model except for defensive blocks, individual attacking actions, and passing tempo. For the remaining variables, as shown in Table 2 and the error bars of Figure 2 and Figure 3, the probability of winning is higher for teams that choose: (1) the possession style compared to teams that choose the direct style in the build up; (2) to perform counterattacks rather than positional attacks in attacking transition; (3) to prevent opponents’ counterattacks, leading them to positional attacks in defensive transition; (4) to create more attacks from open play compared to attacks from set pieces; (5) to make fewer crosses; (6) to not allow opponents to create attacks from open play, limiting them to attacks from set pieces; (7) to prefer high press instead of deep press; (8) to perform many individual defending actions; (9) to create more attacks through the center compared to the flanks; (10) to be able to create final attempts even with a small percentage of ball possession; (11) to avoid fouls and yellow cards; (12) to attack aggressively; and (13) to not frequently adopt the offside trap. The model, overall, correctly predicted 81.9% of the cases.

## 4. Discussion

Our study unveils that adopting specific playing styles in various tactical situations significantly influences a match’s outcome, marking a pivotal advance in performance analysis within football. These findings underscore the critical importance of integrating tactical aspects as key performance indicators (KPIs), a dimension previously underexplored. This research not only highlights the novelty and significance of examining football through a tactical lens, but also sets a precedent for future studies, offering a comprehensive framework that could revolutionize tactical planning and in-game strategy, ultimately enhancing a team’s likelihood of success.

### 4.1. Ball Possession Phase

In the domain of football performance analysis, the tactical approach to build-up play has emerged as a significant factor in the success of winning teams, a fact that is also confirmed in our own research. Empirical studies [2,13,14,16,17,20,21,34,35,36] converge on the assertion that possession-style play correlates positively with the probability of winning. Winning teams have been consistently observed to maintain higher percentages of ball possession, and successfully complete a greater number of passes, which aligns with the understanding that possession can create more goal-scoring opportunities. The possession style, characterized by controlled ball circulation and strategic passing sequences, appears to enable teams to exploit opponent weaknesses and apply their strengths effectively [36]. It allows for a structured phase of play, initiating from defenders and involving all players in orchestrated movements aimed at disrupting organized defenses. The empirical evidence points to the direct impact of ball possession and short passes on the likelihood of achieving victory [17], while the meticulous organization of build-up play is a testament to a team’s tactical proficiency [36]. Notwithstanding this, Liu, Hopkins and Gómez [16] underscore the complexity of the possession–success relationship, highlighting potential negative within-team effects, suggesting that the contextual application of ball possession and passing accuracy is crucial. This nuanced perspective is supported by Pratas, Volossovitch and Carita [35], who emphasize the dependence of possession and direct playing styles’ effectiveness on match-specific factors, team capabilities, and the adaptive application of these styles. In summary, contemporary research advocates for a tactical approach where possession style in the build-up phase is not only associated with higher probabilities of scoring, but also serves as a vital strategic tool in the arsenal of successful football teams. It accentuates the merit of possession-based tactics while also recognizing the need for adaptable strategies that respond to the fluid dynamics of match situations.

Regarding the remaining variables concerning the possession phase, our research points to a shift in tactical preferences towards central play, with a high tendency to create final attempts and a de-emphasis on crossing for successful teams. The analysis of match outcomes reveals that teams with a higher probability of winning tend to engage in fewer crosses, focusing instead on direct central attacks and the ability to create shots on goal with minimal possession, as supported by empirical studies [13,16,17,37,38,39]. The declining emphasis on crosses as a mechanism for goal creation is evidenced by the negative correlation between crossing frequency and winning outcomes. Studies consistently show that while crosses constitute a substantial proportion of assists, goals are more frequently the result of plays initiated from central and advanced areas, where collective tactical play overshadows individual efforts [40]. This transition to a more central focus in attack aligns with the tactical evolution of football, where maintaining possession in central areas can facilitate quicker penetration into shooting zones [37]. In addition, individual attacking actions, such as dribbles, appear to have a less significant impact on the probability of winning, suggesting that while advanced individual skills can enhance team performance, they do not necessarily increase the effectiveness of converting opportunities into goals when overemphasized [15,41]. Instead, strategic, centrally coordinated team play seems to yield more effective goal-scoring opportunities, with research affirming the positive effects of shots and shots on target on match outcomes [13,14,19]. Furthermore, the study indicates that adopting a fast passing tempo is not universally beneficial. Its effectiveness is highly situational and may be compromised by external factors such as playing away or facing stronger opposition, which necessitates adaptability in playing styles [42]. Teams that have succeeded in dynamically adjusting their tactics in response to the unfolding match context have shown greater efficacy in securing victories. In essence, the contemporary football landscape appears to reward teams that demonstrate a tactical inclination towards central attacks, efficient creation of final attempts with economical possession, and adaptable strategies that capitalize on the specific conditions of each match. This analytical insight into build-up play and related tactical situations serves as a vital guideline for teams aiming to enhance their competitive edge.

### 4.2. Opponent’s Ball Possession Phase

In modern football, defensive strategies are just as crucial as offensive tactics, and the phase of play when the opponent has the ball is a significant determinant of match outcomes. Our research provides compelling evidence that certain defensive behaviors are strongly correlated with winning. High-pressing, characterized by attempting to recover possession near the opponent’s goal, significantly increases the chances of victory. This aggressive strategy aligns with the conclusions of the reviews carried out by Pratas, Volossovitch and Carita [35] and González-Rodenas, Malavés, Desantes, Ramírez, Hervás and Malavés [40], which emphasize the advantages of regaining control closer to the opponent’s goal for faster transition into shooting zones. The efficacy of high-press defense has been further substantiated by Low et al. [43], despite acknowledging potential risks associated with this strategy, such as leaving spaces for opponents to exploit. Similarly, Bauer et al. [44] contrast the mid-block and low-block approaches, with the former focusing on structured defense in the midfield and the latter compressing near the goal to prevent shots. The study suggests that the adaptive transition from a mid-block to a low-block reflects a team’s response to escalating pressure, aiming to minimize attackers’ space and reduce the likelihood of conceding goals. In our research, it appeared that the choice between a mid-block and a low-block does not significantly factor into explaining a victorious outcome. This is due to the fact that, on one hand, after regaining possession while in a mid-block, the team is closer to the opponent’s goal. On the other hand, when in a low-block, there is no space behind the defensive line; however, if the ball is regained, there is more space behind the opponent’s defensive line. Regarding individual defensive actions, our research aligns with Liu, Hopkins and Gómez [16], underscoring the effectiveness of tackles and defensive challenges in contributing to winning outcomes. However, it also cautions against aggressive behaviors that lead to fouls and yellow cards, as these can adversely affect a team’s performance. Fernández-Cortés, Gómez-Ruano, Mancha-Triguero, Ibáñez and García-Rubio [13] support this finding, showing that winners typically accrue fewer fouls and cards. Furthermore, Badiella et al. [45] discuss the strategic implications of yellow cards on a team’s defensive approach and the need for coaches to carefully manage players at risk of suspension. It is evident that while many individual defensive actions are conducive to winning, they must be balanced with a disciplined approach to avoid unnecessary fouls and cards. The tactical decision between high-press and deep-press defense must be informed by a team’s strengths, the match context, and the dynamics of the opposition. Winning teams excel not just through their ability to attack but through strategically calculated defensive play that maximizes possession recovery and minimizes disciplinary setbacks, leading to a robust and effective approach to the beautiful game.

### 4.3. Transitions

Transitional phases in football, namely attacking and defensive transitions, play a pivotal role in a team’s success. Our research underlines the critical importance of counterattacking strategies. In attacking transitions, the probability of winning is heightened for teams that utilize swift counterattacks over positional play. This preference for counterattacks is supported by González-Rodenas, Malavés, Desantes, Ramírez, Hervás and Malavés [40], who point out their higher effectiveness in creating goal-scoring opportunities compared to positional attacks. The comprehensive review of Eusebio et al. [46] further elucidates the definition and strategic imperative of counterattacks, emphasizing their speed and ability to exploit defensive imbalances. The potent impact of counterattacks is validated by Liu, Gomez, Lago-Peñas and Sampaio [17], highlighting the positive effects of shots from counterattacks on winning probabilities. Lopez-Valenciano et al. [47] echo this sentiment, advocating for rapid transitions from defense to offense as a key component for successful outcomes. The findings of Tenga et al. [48] corroborate this, indicating a higher goal-scoring efficiency for counterattacks than elaborate attacks. Sarmento et al. [49] quantify this efficiency, noting a 40% increase in the success of offensive sequences when counterattacking tactics are employed. On the defensive side, the study by Gonzalez-Rodenas et al. [50] reveals the dangers of not applying immediate pressure after losing ball possession, as it substantially increases the chances of conceding counterattacking opportunities. The effectiveness of a counterattacking style is particularly pronounced when teams are in the lead, as indicated by Fernández Navarro [39]. The triumph of Leicester City, as analyzed by Gollan et al. [51], serves as a case study for the championship-winning potential of teams proficient in playing transitions, highlighting the importance of speed, technical ability, anticipation, and game intelligence. In summary, our research and the corroborating literature illustrate that counterattacks in both attacking and defensive transitions are instrumental for football teams aiming to enhance their winning prospects. These findings encourage a tactical approach that is nimble, perceptive, and attuned to the ever-changing dynamics of the game.

### 4.4. The Paradox of Offsides

Interestingly, our research indicates that teams caught offside more frequently (attacking aggressively) increase their chances of winning, while teams that manage to catch opponents in the offside trap have reduced chances of victory. This does not seem logical since being caught offside interrupts a team’s attack and results in the ball being given to the opponent. Nonetheless, all previous studies that have dealt with this factor agree with our results. Specifically, Zhou, Zhang, Lorenzo Calvo and Cui [19] and Fernández-Cortés, Gómez-Ruano, Mancha-Triguero, Ibáñez and García-Rubio [13] found that winning teams are caught offside more frequently compared to losing ones. A possible explanation for this phenomenon is that winning teams have more ball possession and create more attacks. Additionally, teams frequently caught offside may appear to be taking risks, but this possibly reflects a more aggressive and dynamic gameplay, which may create more goal-scoring opportunities. Similarly, our research found that attacks from open play increase the likelihood of victory compared to attacks from set pieces, even though set pieces are much fewer than open-play attacks [40] yet account for 30–40% of goals [52]. A possible explanation for this is that InStat Scout registers as attacks only the ball possessions where a player receives the ball in the opponent’s half, excluding ball possessions that are interrupted before the ball crosses the midfield. Furthermore, the fact that throw-ins (which are the most frequent among set pieces but also have the lowest success rate) are included in the set pieces might “unfairly” represent the other types of set pieces. Perhaps normalizing the instances of offside relative to the number of a team’s attacks, as well as examining each type of set piece separately, taking into account all ball possessions and not just the definition used by InStat Scout for attacks, could lead to different results. Therefore, the effectiveness of specific play strategies, such as offside tactics and set pieces, should be evaluated in light of the diverse methodological approaches and data definitions employed across studies. Only through such rigorous examination can we hope to understand the true impact of these strategies on match outcomes.

### 4.5. Limitations

Despite the innovative insights our research provides, it is important to acknowledge certain limitations. The study’s reliance on available datasets from specific European championships might not capture the full diversity of playing styles and tactical situations present in global football contexts. Furthermore, this research utilizes a static analytical approach that, despite its widespread application, only offers a momentary glimpse into performance, neglecting the fluid nature of football matches [35]. Such a method may fail to recognize the complex interplay among players and the evolving circumstances of the game, possibly omitting vital details. This fixed viewpoint differs from dynamic analysis, which accounts for the state of play at every moment, delivering a fuller insight into performance trends and results [53]. Nevertheless, in spite of its shortcomings, the static approach remains the predominant method in the study of football performance analysis, yielding valuable insights for coaches [2,35]. Furthermore, although InStat Scout (from which the data for the original research were obtained) is recognized for its reliability as a data source [54,55], inconsistencies in the accuracy and consistency of data entry across various countries and leagues may introduce biases. These variations in data collection methods could influence the study’s findings, indicating a potential for inconsistency. Lastly, while the predictive accuracy of our model is high, it represents a snapshot within the constantly evolving landscape of football tactics, necessitating continuous validation and refinement to maintain its relevance and applicability. Moreover, adding situational variables to the model may increase its predictive ability even more.

## 5. Conclusions

Our investigation into tactical situations and playing styles in football has yielded substantive findings with practical implications. The most salient outcome indicates that possession style in the build-up phase, the effective utilization of counterattacks during attacking transitions, and an aggressive yet cautious defensive posture significantly enhance a team’s probability of winning. These findings provide empirical support for the integration of tactical nuances as KPIs, thus filling a gap in the existing literature and offering a more intricate understanding of the determinants of match success. For practitioners, specifically coaches and analysts, our results underscore the importance of developing strategies that prioritize ball control, enable swift transitions from defense to attack, and employ a high press while mitigating risks associated with aggressive defense. These strategies should be tailored to the team’s strengths and consider the opposition’s tactical setup.

The scientific and sports community can utilize these analysis models to enhance training sessions and improve team performance. To favor possession-style play, coaches can design drills that emphasize ball control, short passing sequences, and maintaining possession under pressure, such as rondo exercises and small-sided games, which improve players’ ability to retain the ball and make quick, accurate passes [56,57]. Additionally, tactical sessions can focus on building up play from the back, involving all players in orchestrated movements to enhance team coordination and positioning. To counteract offensive models, developing defensive organization through structured defensive blocks, both mid-block and low-block formations, can be beneficial, along with pressing drills that teach players to regain possession quickly near the opponent’s goal through coordinated pressing triggers and recovery runs [58]. For developing defensive behaviors, training should include individual defending drills that emphasize tackling, intercepting passes, and winning duels, as well as transition defense practices where players quickly reorganize and apply pressure after losing possession [59]. Enhancing counterattacking strategies involves training players to transition quickly from defense to attack, with minimal touches and maximum speed to exploit defensive gaps, and improving decision-making during counterattacks through video analysis and situational drills to ensure optimal choices in creating scoring opportunities [60,61].

The contribution of our research to the international body of literature is multifaceted. It provides one of the most comprehensive analyses of tactical KPIs, utilizing an extensive dataset across multiple European leagues. By demonstrating the significant role of tactical situations in the outcome of a match, this research sets the groundwork for future investigations. We suggest that subsequent studies could delve deeper into the contextual factors influencing the effectiveness of the identified tactical approaches. Such research could examine how variables like match location, opponent strength, and match dynamics influence tactical efficacy. Additionally, longitudinal studies tracking the evolution of playing styles could offer insights into the dynamic nature of football tactics. The goal is to continually refine the understanding of how tactical situations influence game outcomes, paving the way for innovative and adaptable football strategies.

## Figures and Tables

**Table 1:** Tactical situations along with the corresponding styles.

Tactical Situation (Latent Variables)	Playing Styles	
	High Values of the Factor Scores	Low Values of the Factor Scores
Build up	Possession style	Direct style
Attacking transition	Counterattack	Positional attack
Defensive transition	Opponent’s counterattack	Opponent’s positional attack
Type of attack	Set pieces attack	Open play attack
Crosses	Many crosses	Few crosses
Type of opponent attack	Opponent’s open play attack	Opponent’s set pieces attack
Defensive blocks	Mid-block	Low-block
Press	High press	Deep press
Individual defensive actions	Many individual defending actions	Few individual defending actions
Width of creative phase	Center attack	Wide attack
Individual attacking actions	Many individual attacking actions	Few individual attacking actions
Creating final attempts	Little possession required to generate final attempts (strong tendency)	High possession required to generate final attempts (low tedency)
Passing tempo	Low passing tempo	High passing tempo
Defending aggressively	Low defensive aggressiveness	High defensive aggressiveness
Attacking aggressively	High attacking aggressiveness	Low attacking aggressiveness
Offside trap	More frequent adoption of the offside trap	Less frequent adoption of the offside trap

**Table 2:** Parameter estimates for predictors of RESULT.

Parameter	B	Wald Chi-Square	Sig.	Exp (B)
(Intercept)	−0.65	207.71	0.00	0.52
Build up	0.38	83.21	0.00	1.46
Att. transition	0.18	31.61	0.00	1.19
Def. transition	−0.25	60.86	0.00	0.78
Type of attack	−0.21	39.93	0.00	0.81
Crosses	−0.43	150.53	0.00	0.65
Type of opp. attack	−0.12	13.74	0.00	0.89
Def. blocks	−0.05	2.68	0.10	0.95
Press	0.13	17.30	0.00	1.14
Ind. def. actions	0.07	5.44	0.02	1.08
Width of creative phase	0.08	5.06	0.02	1.08
Ind. att. actions	−0.04	1.52	0.22	0.96
Creating final attempts	0.80	430.49	0.00	2.22
Passing tempo	0.02	0.58	0.45	1.02
Def. aggressively	0.12	11.02	0.00	1.12
Att. aggressively	0.42	154.74	0.00	1.52
Offside trap	−0.19	37.16	0.00	0.83

## Data Availability

The data presented in this study are available on request from the corresponding author.
